# Complete Genome Sequence of IME207, a Novel Bacteriophage Which Can Lyse Multidrug-Resistant *Klebsiella pneumoniae* and *Salmonella*

**DOI:** 10.1128/genomeA.01015-16

**Published:** 2016-10-27

**Authors:** Yannan Liu, Liyuan Mi, Zhiqing Mi, Yong Huang, Puyuan Li, Xianglilan Zhang, Yigang Tong, Changqing Bai

**Affiliations:** a Department of Respiratory and Critical Care Diseases, 307th Hospital of PLA, Beijing, China; b State Key Laboratory of Pathogen and Biosecurity, Beijing Institute of Microbiology and Epidemiology, Beijing, China; c College of Life Sciences, Inner Mongolia Agricultural University, Huhhot, Inner Mongolia, China

## Abstract

A novel lytic *Salmonella* bacteriophage was isolated by using *Klebsiella pneumoniae* as host cells. The phage’s genome was determined to be 47,564 bp and has the highest similarity to *Salmonella* phage E1 and *Salmonella* phage 64795_sal3, with coverages of 61% and 56%, respectively. Here, we announce the phage’s complete genome.

## GENOME ANNOUNCEMENT

The genus *Salmonella* is composed of Gram-negative facultative anaerobic bacilli classified within the family *Enterobacteriaceae* (1). *Salmonella* infections are a major cause of acute gastroenteritis and septicemia (2), and there are many infective routes, such as from feed to animal, from animal to animal, or from human to animal (3). In recent years, with the abuse of antibiotics in humans and animals, more and more multidrug-resistant *Salmonella* strains have been emerging (4–7). Therefore, the development of alternatives to conventional antibiotics is imperative, and the use of bacteriophages which infect and lyse bacteria is one of the significant options for controlling bacterial infections (8). Here, we isolated a novel lytic *Salmonella* bacteriophage IME207 from hospital sewage using a multidrug-resistant *Klebsiella pneumoniae* as the host, and the phage IME207 can lyse strains of *Klebsiella pneumoniae* and *Salmonella*.

Genomic DNA of *Salmonella* phage IME207 was extracted with the proteinase K-SDS method (9). The genomic DNA of phage was subjected to high-throughput sequencing using the Ion Personal Genome Machine (Life Technologies, USA). A total of 383,580 raw reads were obtained, with a mean read length of 282 bp. A total of 377,112 reads were assembled into a complete genome using the Newbler 2.9.1 software, and the coverage ranges from 890× to 3,056×. The complete genome was annotated using the online tool RAST, while sequence similarity analysis was performed using NCBI BLAST.

NCBI BLAST analysis indicated that the complete genome sequence of phage IME207 has high similarity to those of *Salmonella* phage E1 (GenBank accession number AM491472.1) and *Salmonella* phage 64795_sal3 (GenBank accession number KX017520.1), with coverages of 61% and 56%, respectively. The genomic length of phage IME207 was 47,564 bp, with a G+C content of 46.4%. The annotation results of the RAST online tool predicted that phage IME207 contained 94 putative open reading frames (ORFs), with 58 ORFs located on the positive strand. The total length of the 94 ORFs was 44,174 bp, and the length of each ORF varied from 113 bp to 2,930 bp, resulting in a coding density of 92.9% (total ORF length/total genome length). Of the 94 ORFs of phage IME207, only 21 ORFs were predicted to encode proteins with known functions. The products of the 21 ORFs belong to phage structure and packaging module (containing neck whisker protein, tape measure protein, tail fiber protein, head decoration protein, coat protein, inner membrane and outer membrane spanin protein), replication/transcription module (containing transcriptional regulators, homing endonuclease, single-stranded DNA-binding protein, recombinase, exonuclease, DNA primase/helicase, terminase large subunit, and DNA polymerase III beta subunit), and host lysis module (containing lysin and holin protein). In conclusion, we isolated a novel bacteriophage which can lyse *Klebsiella pneumoniae* and *Salmonella*. Genomic analysis showed that the phage genome did not encode integrase or contain toxin genes or any transposon elements, which means that the phage has a potential to be developed as an alternative antimicrobial agent.

### Accession number(s).

The complete genome sequence of *Salmonella* phage IME207 was deposited in GenBank under the accession number KX523699.
